# Performance comparison of dwarf laying hens segregating for the naked neck gene in temperate and subtropical environments

**DOI:** 10.1186/1297-9686-41-13

**Published:** 2009-01-16

**Authors:** Chih-Feng Chen, David Gourichon, Nein-Zu Huang, Yen-Pai Lee, André Bordas, Michèle Tixier-Boichard

**Affiliations:** 1 Department of Animal Science, National Chung-Hsing University, 40227 Taichung, Taiwan; 2 INRA, Unité expérimentale PEAT, 37380 Nouzilly, France; 3 INRA, AgroParisTech, UMR1236 Génétique et Diversité Animales, 78350 Jouy-en-Josas, France

## Abstract

This study compares laying performances between two environments of dwarf laying hen lines segregating for the naked neck mutation (*NA *locus), a selected dwarf line of brown-egg layers and its control line. Layers with one of the three genotypes at the *NA *locus were produced from 11 sires from the control line and 12 sires from the selected line. Two hatches produced 216 adult hens in Taiwan and 297 hens in France. Genetic parameters for laying traits were estimated in each environment and the ranking of sire breeding values was compared between environments. Laying performance was lower, and mortality was higher in Taiwan than in France. The line by environment interaction was highly significant for body weight at 16 weeks, clutch length and egg number, with or without Box-Cox transformation. The selected line was more sensitive to environmental change but in Taiwan it could maintain a higher egg number than the control line. Estimated heritability values in the selected line were higher in France than in Taiwan, but not for all the traits in the control line. The rank correlations between sire breeding values were low within the selected line and slightly higher in the control line. A few sire families showed a good ranking in both environments, suggesting that some families may adapt better to environmental change.

## Introduction

In poultry selection programmes, birds are generally raised in a uniform environment to record the selected traits and evaluate genetic values. Thus, most commercial populations are obtained from breeding farms with a controlled environment and are delivered to production farms with variable environments across the world. As a consequence, genotype × environment (GxE) interactions may occur. Under subtropical environments, growth rate as well as egg production are generally depressed by high ambient temperature [1,2]. Introduction of some major genes, such as the naked neck (*NA*) gene, in chicken lines can be used to alleviate heat stress, as discussed in several studies carried out at high ambient temperatures [1,3-5]. Heat tolerance of laying hens is an important issue for the impact of G × E on egg production because the productive period is long and the impact of heat stress increases with the hens' age [6].

At INRA, a selection experiment was undertaken to improve egg production of brown-egg layers by increasing clutch length, in the presence of two major genes known to improve heat tolerance, the naked neck gene [7] and the sex-linked dwarf gene [8]. After 16 generations, significant genetic progress on egg production was observed in two dwarf lines, one carrying the *NA *gene and one not carrying it [9-11]. The reduction of feather mass due to the *NA *gene is lower in heterozygous birds than in homozygous birds, *i.e. *27% and 22% respectively for heterozygous females and males, and 41% and 33% respectively for homozygous females and males [12]. Heat tolerance is significantly improved by the presence of the *NA *gene and significant genotype by temperature interactions were observed for egg production, egg mass and feed intake under a 32°C constant temperature environment, which represents a severe temperature stress [3]. The purpose of this paper was to study the laying performance in real subtropical conditions of the genotypes obtained from the selection experiment on clutch length, in comparison to the performance in the selection environment. In order to investigate adaptation of hens, genetic parameters for laying traits were estimated in each environment and the ranking of sire breeding values was compared between environments.

## Methods

### Genetic background

Two lines of dwarf brown-egg layers established from a common base population in 1985 have been selected on average clutch length for 16 generations. The selection procedure has been described by Chen and Tixier-Boichard [9]. One line (L1) was normally feathered and the other (L2) was homozygous for the NA*NA mutation; a control line segregating for the NA*NA mutation was also maintained. In 2001, both selected lines were crossed to produce a new line segregating for the NA*NA mutation, which was maintained with a mild selection pressure on average clutch length. In 2003, 11 males and 44 females from the control line, and 12 males and 48 females from the newly formed selected line, were used to generate individuals with the three possible genotypes at the *NA *locus *i.e. *normally feathered (NA*N/NA*N), heterozygous carriers of the naked neck mutation (NA*NA/NA*N) or homozygous carriers (NA*NA/NA*NA). Chicks were obtained in two hatches at a three-week interval for rearing in Taiwan and France, respectively.

### Husbandry

#### Taiwan

**Figure 1:**
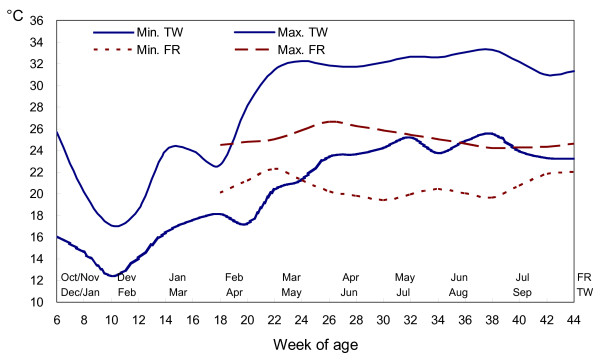
**The average maximum and minimum ambient temperature by two weeks in Taiwan (TW) and during the laying period in France (FR)**. (average temperature, 22 ± 1°C, during the rearing period in France).

On November 28, 2003, one-day-old chicks were imported to Taiwan. After a 10-day quarantine, they were transferred to the experimental farm of the National Chung-Hsing University. All the chicks were debeaked and reared in floor pens up to 16 weeks. Chicks were vaccinated against the following diseases Marek's disease, fowl pox, Newcastle disease, infectious bronchitis, infectious bursal disease, respiratory enteritic orphan, infectious laryngo-tracheitis, infectious coryza, avian encephalomyelitis and egg drop syndrome. At 17 weeks of age, pullets were housed in individual cages in open house, supplied with ground water and reared in natural daylight. The length of daylight increased gradually from 10.5 to 12 h between ages 4 and 16 weeks and then from 17 weeks of age till the end of the test, a fixed light regimen 14L:10D was applied. This lighting regimen had been used throughout the selection experiment in France and was taken as the reference lighting regimen for the laying period. Water and food were supplied *ad libitum*, layer mash contained 18.0% CP and 11.5 MJ ME/kg and temperature and relative humidity were monitored continuously. Figure 1 shows the curve of daily maximum and minimum temperatures in the chicken house.

#### France

On September 18, 2003, full-sibs were reared in the experimental farm of INRA, Tours. All chicks were reared in floor pens up to 16 weeks. Chicks were vaccinated against the following diseases: Marek's disease, infectious bronchitis, Gumboro, Newcastle disease and avian encephalomyelitis. At 17 weeks of age, pullets were housed in individual cages in a windowless house with light regimens fixed as 10L:14D and 14L:10D in growth and laying periods, respectively. This lighting regimen had been used since several years for experimental lines in France and was taken as the reference lighting regimen for the laying period. Water and food were supplied *ad libitum*. Monthly averages of maximal and minimal temperatures were 25 ± 1°C and 21 ± 1°C, respectively (February to July/18 to 44 weeks of age). Relative humidity ranged from 60 to 80%. Layer mash contained 16.4% CP and 11.2 MJ ME/kg.

### Variables under study

The egg number (EN) was recorded from the age at first egg (AFE) to 44 weeks of age. For each hen, the laying rate (LR) was obtained from the ratio of egg number (whatever the egg status) to the number of days since the first egg. A break of one day at least between ovipositions was taken as the end of a clutch. The average clutch length (CL) was calculated as the arithmetic mean of all clutches recorded, from the first egg until 44 weeks of age. In addition, egg weight (EW) was obtained at 34 weeks of age (36 weeks in France), by collecting two eggs per hen laid on consecutive days within a week. The body weights were measured at the entry in the poultry house (BW16) and at adult age (ABW) *i.e. *34 weeks in Taiwan, and 40 weeks in France.

In order to satisfy the classical hypothesis for describing traits with polygenic inheritance via a linear model with normal error, Box-Cox power transformation was used to normalize trait distribution [13,9]. The parameters (t) were found to be -0.214, and 1.40 for the transformed value of average clutch length (TCL) and for transformed value of total egg number (TEN), respectively.

### Statistical analysis

The linear model used for the ANOVA of those traits was as follows:

Y_ijkl _= *μ *+ E_i _+ L_j _+ G_k _+ (E × L)_ij _+ (ExG)_ik _+ (L × G)_jk _+ (E × L × G)_ijk _+ e_ijkl_

where Y_ijkl _= individual observation, *μ *= overall mean, E_i _= fixed effect of environment, i = 1 to 2, L_j _= fixed effect of line, j = 1 to 2, G_k _= fixed effect of the naked neck genotype, k = 1 to 3, (E × L × G)_ijk _= fixed effect of the third order interaction between all fixed effects, (E × L)_ij _= fixed effect of environment-by-line interaction, (E × G)_ik _= fixed effect of genotype-by-environment interaction, (L × G)_jk _= fixed effect of line-by-genotype interaction, and e_ijkl _= random error. All analyses were carried with the GLM procedure of the SAS^® ^program library [14]. Means were considered significantly different at P < 0.05.

### Genetic analysis

Variance and covariance components were estimated using the Restricted Maximum Likelihood (REML) procedure with the VCE package of Groeneveld [15]. The following animal model was applied to all traits on the whole data set:

*Y*_*ij *_= *μ *+ *G*_*i *_+ *a*_*j *_+ *e*_*ij*_

where *Y*_*ij *_= individual observation for traits, *μ *= overall mean, *G*_*i *_= fixed effect of genotype at the *NA *locus, *a*_*j *_= random animal effect and *e*_*ij *_= random error. Expectation and variance of the vector of performance, *y*, were distributed as follows, in matrix notation:

$$\begin{array}{ccc} E\left[\begin{array}{c} y\\ a\\ e \end{array}\right]=\left[\begin{array}{c} X\beta \\ 0\\ 0 \end{array}\right] & \text{and} & V\left[\begin{array}{c} a\\ e \end{array}\right]=\left[\begin{array}{cc} A⊗G & 0\\ 0 & \overset{k}{\underset{j=1}{⊕}}R_{j} \end{array}\right] \end{array}$$

where *y *= observed performance, *a *= individual additive genetic value, e = residual, *β *= vector of genotype fixed effect, *X *= incidence matrix of vector *β*, *A *= numerator relationship matrix; and *G *= variance-covariance matrix for the additive genetic effect; *k *= the number of records, and *Rj *= residual variance-covariance matrix for animal *j*. The direct product and direct sum of matrices are indicated by ⊗ and ⊕, respectively.

In order to estimate the genetic correlation between performance measured in the two environments, traits measured in France (F) and Taiwan (T) were treated as distinct BW16_T _and BW16_F_, the subscript indicating the environment. Because a significant environment-by-line interaction was observed for most of the traits, multi-trait analyses were performed separately for each line. It was not possible to analyse all the traits together because of large computing requirements. Therefore eight analyses with four traits each were implemented in the present study. For instance, concerning body weight traits, one four-trait analysis (BW16_F_, BW16_T_, ABW_F_, ABW_T_) was performed in each line.

Sire breeding values were evaluated in each environment for each trait by best linear unbiased prediction (BLUP) using a mixed linear model with the PEST package [16], with the same model described above. A Spearman correlation analysis was used to compare the sire breeding values obtained either in France or in Taiwan.

## Results

Total mortality in Taiwan was 22.8% and 23.6% for control and selected lines, respectively, whereas it was below 5% in France. There was no occurrence of epidemic infectious disease in either country. In Taiwan, a hot foehn wind caused a rise in temperature up to 40.3°C during 7 h on July 1^st^. On that day, mortalities were 6.2%, 13.6% and 5.5% for NA*N/NA*N, NA*NA/NA*N and NA*NA/NA*NA genotypes, respectively. During that week, cumulative mortalities were 7.8%, 14.6% and 9.4% for each of the three genotypes, respectively. Finally, the experiment included 297 hens in France and 216 hens in Taiwan. The proportion of hens carrying the homozygous NA*NA/NA*NA genotype was slightly lower in Taiwan (20–22%) as compared to France (25%).

### Effects of environment, line, genotype and interactions

The third order interaction between main effects was never significant, although it was at the limit of significance for egg weight (P < 0.10). There were no significant interactions between the naked neck genotype and either the line or the environment (Table 1).

**Table 1:** Results of the analysis of variance for the performance of laying hens according to environment (France or Taiwan) to selection background (selected line or control line) and to the naked neck genotype (homozygous normal, heterozygous or homozygous for the naked neck mutation)

Traits	Environment (E)	Line (L)	Genotype (G)	E × L	G × E	G × L	G × E × L
Body weight at 16 weeks	***	***	*	***			
Age at first egg	***	***	*				
Clutch length	***	***		***			
Clutch length (Trans.)	**	***		***			
Egg number at 44 weeks	***	***		*			
Egg number at 44 weeks (Trans.)	***	***		***			
Laying rate	***	***					
Adult body weight	***	***					
Egg weight	***	*	*				+

Significant at +, P < 0.1; *, P < 0.05; **, P < 0.01; *** P < 0.001

The line by environment interaction was highly significant for BW16, clutch length and egg number, with or without Box-Cox transformation (Table 1). Body weight differed between lines in Taiwan to a larger extent than in France: the decrease in body weight observed in Taiwan as compared to France was stronger for the selected line. Clutch length and egg number were smaller in Taiwan and to a larger extent for the selected line *i.e. *the decrease in average clutch length, the selected trait, was 26.6% (3.19 vs. 2.34) and 59.4% (13.89 vs. 5.64) in the control and the selected lines, respectively. However, performance of the selected line still remained much higher than that of the control line in both environments.

The naked neck genotype had a significant effect on body weight at 16 weeks (BW16), age at first egg (AFE) and egg weight (EW) (Table 1). Homozygous naked neck hens had a lower BW16 and a higher AFE, except in the control line in France. They also exhibited a larger EW, but the difference was significant only for the control line in France, and for the selected line in Taiwan (Tables 2 and 3). Thus, the *NA *gene limited the negative impact of the change in environment on egg weight in the selected line.

**Table 2:** Performances of egg production in France

	Control line			Selection line		
	
Traits	NA*N/NA*N ^1^	NA*NA/NA*N	NA*NA/NA*NA	NA*N/NA*N	NA*NA/NA*N	NA*NA/NA*NA
(Number of birds)	(20)	(56)	(31)	(43)	(93)	(44)
Body weight at 16 weeks, g	1420 ± 37^2^	1435 ± 16	1443 ± 33	1457 ± 21 ^a^	1441 ± 18 ^a^	1371 ± 18 ^b^
Age at first egg, day	139.5 ± 4.0	135.5 ± 1.2	139.4 ± 1.7	130.1 ± 1.2	133.8 ± 2.1	132.8 ± 1.2
Clutch length, egg	2.93 ± 0.24	3.32 ± 0.18	3.11 ± 0.20	13.35 ± 1.76	13.67 ± 0.86	11.93 ± 0.92
Clutch length (Trans.)	6.69 ± 0.50	7.38 ± 0.29	7.04 ± 0.40	14.01 ± 0.32	13.96 ± 0.30	13.62 ± 0.36
Egg number at 44 weeks	111.5 ± 8.1	123.0 ± 3.9	120.1 ± 5.0	167.4 ± 1.6	163.6 ± 2.8	157.8 ± 4.1
Egg number at 44 weeks (Trans.)	81.4 ± 7.1	92.0 ± 3.6	88.9 ± 4.8	139.5 ± 1.9	136.2 ± 2.7	129.5 ± 4.1
Laying rate, %	63.0 ± 4.4	68.9 ± 2.1	68.8 ± 2.7	91.0 ± 0.8	90.3 ± 1.2	87.0 ± 2.1
Adult body weight, g	2156 ± 71	2141 ± 39	2181 ± 67	2071 ± 27 ^a^	2004 ± 26 ^ab^	1924 ± 36 ^b^
Egg weight, g	49.8 ± 1.1 ^b^	51.8 ± 0.6 ^ab^	52.4 ± 0.8 ^a^	51.0 ± 0.6	51.2 ± 0.5	50.3 ± 0.5

^1 ^NA*N/NA*N: normally feathered; NA*NA/NA*N: heterozygous naked neck; NA*NA/NA*NA: homozygous naked neck

^2 ^The values are mean ± standard error

^a, b^Different superscripts indicate significant difference within line at a given environment

**Table 3:** Performances of egg production in Taiwan

	Control line			Selection line		
	
Traits	NA*N/NA*N ^1^	NA*NA/NA*N	NA*NA/NA*NA	NA*N/NA*N	NA*NA/NA*N	NA*NA/NA*NA
(Number of birds)	(25)	(36)	(16)	(49)	(59)	(31)
Body weight at 16 weeks, g	1360 ± 38^2,^^a^	1295 ± 32 ^ab^	1261 ± 21 ^b^	1213 ± 23 ^a^	1213 ± 19 ^a^	1143 ± 24^b^
Age at first egg, day	157.6 ± 2.6 ^a^	160.7 ± 2.8 ^ab^	167.4 ± 3.1 ^b^	153.4 ± 2.0	152.2 ± 1.4	157.4 ± 2.6
Clutch length, egg	2.60 ± 0.17	2.27 ± 0.11	2.09 ± 0.15	5.53 ± 0.40	6.50 ± 0.52	5.46 ± 0.62
Clutch length (Trans.)	6.11 ± 0.35	5.30 ± 0.28	4.81 ± 0.44	9.85 ± 0.39	10.34 ± 0.42	9.68 ± 0.51
Egg number at 44 weeks	80.8 ± 5.2	76.8 ± 4.1	76.7 ± 4.9	112.4 ± 3.7	110.9 ± 4.1	107.4 ± 3.5
Egg number at 44 weeks (Trans.)	51.7 ± 4.3	48.2 ± 3.4	47.5 ± 4.1	81.1 ± 3.4	80.3 ± 3.7	75.5 ± 3.4
Laying rate, %	53.7 ± 3.3	52.3 ± 2.6	54.4 ± 3.1	72.6 ± 2.1	71.0 ± 2.5	71.4 ± 2.2
Adult body weight, g	1720 ± 42	1748 ± 41	1677 ± 36	1587 ± 28	1585 ± 25	1551 ± 37
Egg weight, g	48.3 ± 0.5	49.5 ± 0.7	49.6 ± 0.9	46.6 ± 0.4 ^b^	47.2 ± 0.5 ^b^	49.3 ± 0.6 ^a^

^1 ^NA*N/NA*N: normally feathered; NA*NA/NA*N: heterozygous naked neck; NA*NA/NA*NA: homozygous naked neck

^2 ^The values are mean ± standard error

^a, b^Different superscripts indicate significant differences within lines at a given environment

All traits were significantly influenced by environment and line. For all traits, the performance measured in France was better than the performance obtained in Taiwan. In France, the mean values for CL, EN and LR were respectively 3.19 (untransformed), 120 (untransformed), and 66.9 in the control line and 13.17 (untransformed), 163 (untransformed), and 89.4 in the selected line. In Taiwan, the average clutch length was 2.34 and 5.64 (untransformed values) for the control and selected lines, respectively. After normalization by Box-Cox transformation, the relative decrease of average clutch length in Taiwan was similar for both lines and close to -30% except for the normally feathered hens in the control line, but, in absolute values, the decrease was larger in the higher performing line After normalization, the total egg number in Taiwan as compared to France was even more reduced (by about 40%) as a result of the increase in age at first egg observed in Taiwan (by about 15%). Laying rate was reduced by 20% in Taiwan in both lines.

**Figure 2:**
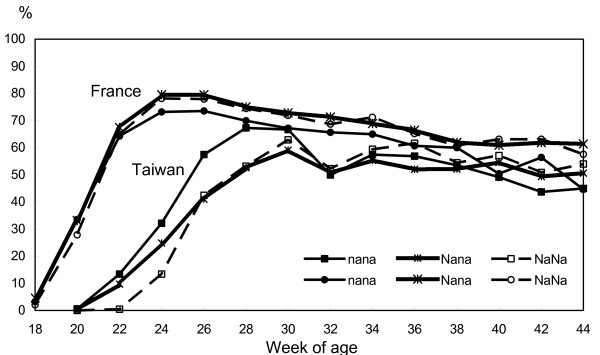
**Hen-day laying rate by two weeks for three genotypes of the control line in France and Taiwan**.

**Figure 3:**
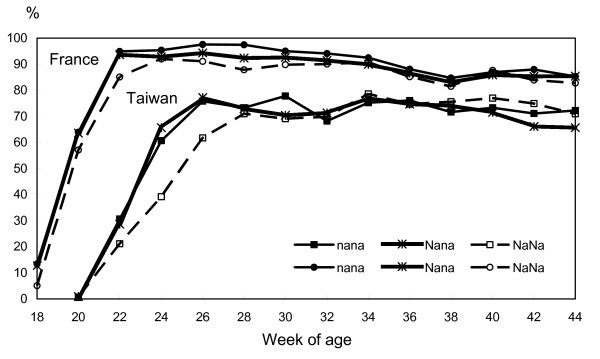
**Hen-day laying rate by two weeks for three genotypes of selected lines in France and Taiwan**.

Comparison of the laying curves obtained in France and Taiwan (Figures 2 and 3) showed differences due to a delayed onset of lay, a lower peak of egg production with a slightly better persistency of lay until the age of 44 weeks. There was a three-week delay of the onset of lay for both lines in Taiwan and, in the selected line, the homozygous NA*NA/NA*NA genotype reached the peak of egg production later, but this was not significant in the control line.

Feed consumption was measured between 31 and 34 weeks of age in Taiwan only. There were no significant differences between lines (2163 vs 2267 for control line vs selection line), or among genotypes (2171, 2281, 2219 for NA*N/NA*N, NA*NA/NA*N, NA*NA/NA*NA genotypes).

### Genetic parameters

Estimates of heritability and genetic correlations between traits measured in the two environments are shown in Table 4 for each line. Heritability values estimated for the selected line were generally higher for traits measured in France and the difference was noticeable for body weight, egg number, laying rate and egg weight. Heritability values obtained in Taiwan for the selected line were very low for egg number and laying rate. Since in most cases, the estimated heritability values exhibited larger asymptotic errors for the control line than for the selected line (because of a limited number of animals), the differences in estimates between the two environments were not as remarkable. However, in some cases high values were obtained *i.e. *for normalized clutch length, which showed a higher estimated heritability in Taiwan (0.91) than in France (0.57).

**Table 4:** Estimated heritability for each trait measured in each environment and genetic correlations considering the records of a given performance in both environments (France-Taiwan) as different traits within each line

Traits	*h*^2 ^in France		*h*^2 ^in Taiwan		Genetic correlation between traits defined for each measurement in each environment	
	
	Control line	Selection line	Control line	Selection line	Control line	Selection line
Body weight at 16 weeks	0.51 ± 0.16^1^	0.85 ± 0.15	0.64 ± 0.16	0.46 ± 0.12	0.44 ± 0.25	0.82 ± 0.13
Age at first egg	0.17 ± 0.11	0.49 ± 0.11	0.31 ± 0.14	0.42 ± 0.12	-0.45 ± 0.38	0.16 ± 0.16
Clutch length (trans.)	0.57 ± 0.11	0.20 ± 0.08	0.91 ± 0.13	0.12 ± 0.07	0.98 ± 0.06	0.49 ± 0.45
Egg number at 44 weeks (trans.)	0.49 ± 0.17	0.32 ± 0.12	0.37 ± 0.18	0.01 ± 0.03	0.48 ± 0.09	-0.10 ± 1.48
Laying rate	0.50 ± 0.18	0.27 ± 0.10	0.35 ± 0.16	0.07 ± 0.06	0.23 ± 0.09	0.14 ± 0.58
Adult body weight	0.52 ± 0.14	0.69 ± 0.14	0.38 ± 0.17	0.34 ± 0.13	0.34 ± 0.25	0.96 ± 0.05
Egg weight	0.39 ± 0.09	0.60 ± 0.10	0.18 ± 0.09	0.38 ± 0.10	0.42 ± 0.35	0.86 ± 0.13

^1 ^asymptotic standard error

Genetic correlations between traits measured in the two environments for the selected line were high and not different from 1 for body weight and egg weight (from 0.82 to 0.96); values were lower and much less accurate for egg laying traits, so that it may be suggested that most of these correlations were null; only age at first egg exhibited a reasonably low asymptotic error, supporting the hypothesis that the correlation did not differ from zero. A different picture was observed for the control line: the highest correlation was observed for clutch length in both environments and was very close to 1, whereas correlations obtained for egg number, laying rate and body weight ranged from 0.34 to 0.48. Actually, correlations for body weight, egg weight and age at first egg were rather lower than in the selected line and exhibited the largest standard errors of all estimates for the control line, suggesting that they may not differ from zero.

The Spearman rank correlations of sire breeding values estimated between the two environments are listed in Table 5 together with their probability values. Most of these correlations were not significant but some differences between lines may be pointed out. Correlations for egg production traits (clutch length, egg number) were moderate to high in the control line (0.72 to 0.88) and differed significantly from zero, whereas they did not differ from zero in the selected line. On the opposite, correlations for body weight and egg weight were higher in the selected line (0.48 to 0.70) than in the control line; the correlation was significant for egg weight in the selected line but not in the control line. The correlation for age at first egg was low and non significant in both lines.

**Table 5:** Spearman correlation coefficients between sire breeding values estimated in both environments

Traits	Control line (sire number = 9)	Selected line (sire number = 12)
Body weight at 16 weeks	0.45, P = 0.224	0.50, P = 0.101
Age at first egg	-0.20, P = 0.606	0.07, P = 0.829
Clutch length (Trans.)	0.88, P = 0.002	0.23, P = 0.471
Egg number at 44 weeks (Trans.)	0.72, P = 0.030	-0.21, P = 0.513
Laying rate	0.57, P = 0.112	-0.01, P = 0.966
Adult body weight	0.12, P = 0.765	0.48, P = 0.112
Egg weight	0.47, P = 0.205	0.70, P = 0.011

## Discussion

### Effects of environment, line, genotype and interaction

The effect of environment was quite large. The performances of all traits were lower in Taiwan and mortality was very high for both lines. Since there was no outbreak of infectious disease, this high level of mortality is rather difficult to explain. It may be due to the cumulated effect of several environmental and husbandry factors differing between France and Taiwan, which affected lines to a similar extent. For instance, these lines are never debeaked in France. To our knowledge, it has never been reported that debeaking has an effect on feed intake and behaviour in dwarf brown-egg layers. Since hens were housed in individual cages, debeaking was not expected to influence performance greatly.

A significant interaction between environments and lines was found for laying traits, which indicated that the selected line was more sensitive to environmental changes than the control line for production level. Thus, performance level influenced the adaptation of laying hens. However, it should be noted that, in Taiwan, the selected line maintained a higher performance for laying rate than the control line. Among the main differences between the two environments, ambient temperature, lighting regimen and diet composition are discussed in more detail below.

Concerning ambient temperature, a favourable effect on hens' adaptation was expected with the naked neck gene. Indeed, at a constant ambient temperature of 32°C, the *NA *gene has been shown to limit the negative effect of long-term heat stress on egg production and feed efficiency traits [3], because of its effect on sensible heat loss, which is increased due to lower feather cover [12,17]. In the present study, the average daily minimum and maximum temperatures ranged between 24.1°C to 32.4°C during the laying period. However, there was no significant effect of the naked neck genotype on laying traits and no interaction between naked neck genotype and environment. This suggests that the naturally cycling temperature was not so stressful for laying hens. In broilers, chickens exposed to temperature cycling over a wide range of 24°C – 35°C, exhibit a similar growth rate to that recorded in birds exposed to a constant temperature [18].

Furthermore, the *NA *gene was not expected to influence adaptation to other husbandry factors than temperature, such as lighting or feed composition. Indeed, since all these factors differed between France and Taiwan, the resulting effect of the *NA *gene could not be identical to the effect observed when only the temperature factor was changed. This study suggests that ambient temperature is not the main driving factor of adaptation of layers to subtropical environments in experimental conditions.

Concerning lighting regimen, it has been shown to play a very important role in the onset of sexual maturity. A constant or decreasing amount of daily light will delay sexual maturity in growing birds. Indeed, in the present study, the amount of daily light in Taiwan decreased during the rearing period, which probably explains the three-week delay for the onset of lay observed in both lines. The control line exhibited the highest values for onset of lay, as previously reported [10]. The delay appeared to be longer for the naked neck hens in both lines, which may also be due to the lower body weight observed with this genotype in Taiwan and in France for the selected line only. Chen and Tixier-Boichard [10] have already observed this effect of the naked neck gene on sexual maturity for the same dwarf lines.

Concerning feed composition, the higher protein percentage used in Taiwan is expected to have a positive effect on egg weight [19], but this is not what we observed. Since egg weight is generally strongly correlated to body weight, the negative effect of the subtropical environment on body weight is probably the main explanation for the lower egg weight observed in Taiwan.

### Genetic parameters

Changes in environment affected genetic parameters to a larger extent in the selected line than in the control line. In the selected line, the estimated heritabilities exhibited higher values for performance recorded in France than in Taiwan, which could be explained by the large environmental variability in Taiwan. Coefficients of variation for clutch length, egg number and laying rate were much larger in Taiwan than in France for the selected line except for the homozygous naked neck hens; the same trend was observed, with a lower magnitude, in the control line (Table 6). Coefficients of variation were generally smaller and remained more stable between environments for body weight, egg weight and sexual maturity in both lines (Table 6). The heritability values for AFE (0.49), TEN (0.32) and LR (0.27) obtained for the selected line in France were close to estimates previously published for the same population [10]. For the selected trait TCL and traits strongly correlated with it (egg number and laying rate), heritability values were higher in the control line, whatever the environment, which is consistent with the decrease in genetic variance due to past selection. This suggests that selection to improve laying performance in Taiwan may be difficult in the selected line.

**Table 6:** Coefficients of variation for traits measured in France and Taiwan, for each line and each genotype at the naked neck locus

	Control line						Selection line					
	
Traits	NA*N/NA*N ^1^		NA*NA/NA*N		NA*NA/NA*NA		NA*N/NA*N		NA*NA/NA*N		NA*NA/NA*NA	
	France	Taiwan	France	Taiwan	France	Taiwan	France	Taiwan	France	Taiwan	France	Taiwan
Body weight at 16 weeks	11.7	14.1	8.19	14.9	12.8	6.74	9.48	13.3	12.0	12.1	8.94	11.5
Age at first egg	12.8	8.16	6.4	10.5	6.8	7.33	5.9	9.3	15.4	7.34	5.8	9.02
Clutch length (Trans.)	33.2	28.8	29.6	32.2	**31.7**	**36.9**	**14.7**	**28.2**	**20.7**	**31.9**	**17.7**	**29.1**
Egg number at 44 weeks (Trans.)	39.1	41.8	**28.9**	**42.2**	30.0	34.4	**8.92**	**29.5**	**18.9**	**36.4**	21.1	25.2
Laying rate	31.4	30.8	**23.1**	**30.3**	22.1	22.7	**5.4**	**20.0**	**12.3**	**27.2**	16.4	16.9
Adult body weight	14.7	11.5	13.6	13.7	17.2	8.51	8.39	12.5	12.6	11.9	12.5	13.4
Egg weight	9.79	5.3	8.17	8.82	8.64	7.39	7.45	6.66	8.79	8.10	6.44	6.84

The largest relative changes between environments are indicated in bold characters.

^1 ^NA*N/NA*N: normally feathered; NA*NA/NA*N: heterozygous naked neck; NA*NA/NA*NA: homozygous naked neck

Estimating genetic correlations between traits measured in two environments is one approach to reveal within-line genotype × environment (G × E) interactions [20]. Correlations significantly lower than 1 indicate the occurrence of GxE interactions. Our results indicate that G × E interactions are more important for laying traits than for body weight and egg weight in the selected line but absent for clutch length in the control line. This is consistent with the fact that the highest performing line was more susceptible to environmental change. Concerning age at first egg, very low correlations were observed in both lines, which were similarly affected by the difference in lighting regimen.

Rank correlations between sire breeding values, calculated for 9 and 12 sires from control and selected lines respectively show no correlation for egg production traits in the selected line, and positive but moderate correlations for body weight and egg weight. The situation is almost opposite in the control line, with a significantly positive correlation for egg production traits and low to moderate correlations for body weight and egg weight. Similarly, rank correlation results confirm genetic correlations results. This emphasizes the high impact of GxE interaction on the within-line selection process that should not be ignored in a breeding program. Within the selected line, a few sires (sire 19 and 16) showed a good ranking in both environments for the selected trait, clutch length (Table 7) suggesting that it may be possible to identify some families with a better adaptation capacity to environmental changes.

**Table 7:** Sire breeding values in the selected line, for traits measured in both environments, data are sorted according to the breeding value for clutch length (transformed for normalization) estimated from performance recorded in France

Sire ID	Clutch length (Trans.)		Egg number at 44 weeks (Trans.)		Laying rate		Adult body weight		Egg weight	
	
	France	Taiwan	France	Taiwan	France	Taiwan	France	Taiwan	France	Taiwan
23	1.3375	-0.6509	9.774	-0.3085	4.26	-1.1137	91.14	-2.08	2.8653	0.2951

**19**	**1.1622**	**0.4122**	**5.462**	**0.4112**	**4.242**	**1.4648**	**-95.65**	**65.05**	**0.9138**	**-0.0335**

**16**	**0.4291**	**0.6636**	**1.453**	**0.4402**	**2.64**	**2.681**	**-183.05**	**-86.1**	**-0.5016**	**0.2148**

21	0.4168	-0.6866	9.636	-0.4325	2.453	-2.4114	-28.82	-57.71	1.269	0.5487

12	0.3184	0.6349	-17.522	0.258	-2.653	2.0701	244.85	72.69	5.6541	2.8713

17	0.2211	0.1083	8.155	0.3474	2.263	0.2681	-69.44	-16.66	-5.4449	-2.4796

18	-0.025	0.3935	7.5	0.2238	1.128	0.3332	9.24	38.79	0.0707	-0.7562

15	-0.127	-0.3903	-4.79	-0.2068	-0.565	0.9742	-222.19	-9.06	-2.2545	1.3863

22	-0.2054	0.121	5.669	-0.3079	1.886	-2.2254	2.83	-96.1	-2.3982	-3.7489

13	-1.0237	0.4648	-0.058	0.2109	-1.338	1.1587	143.12	75.12	-2.6192	-0.8452

14	-1.1715	0.0366	-22.879	0.2269	-13.281	0.1669	103.45	-21.82	3.8258	1.5172

20	-1.3385	-1.1083	-2.386	-0.8631	-1.053	-3.3693	4.54	37.88	-1.3587	1.0144

## Conclusion

Breeders regularly mention the importance of GxE interactions within commercial lines, but data obtained for similar families in contrasted environments, as shown here, are generally not published. Use of a control line, derived from the same base population as the selected line, clearly shows that the sensitivity to a new environment, particularly subtropical climate, is increased by past selection. This greater sensitivity is associated with a decrease in heritability of selected traits. Regular testing of a given line across various environments, and integrating such data in the evaluation programme could monitor this impact. Considering the current predictions on future climate changes, this result shows that the components of adaptation to real subtropical conditions need to be better identified in order to anticipate the negative consequences on the climate change. Indeed, the heat tolerance described for naked neck layers in experimental conditions was not found to particularly improve adaptation to the naked neck layers in Taiwan. Identification of a few sire families capable of achieving a good performance in both environments, temperate and subtropical, suggests that selection for improved adaptation capacity may be feasible. However, a large genetic base will be necessary to identify a sufficiently large number of sires to start a selection programme. Other differences between selected lines, not included in the present study, may also have an influence and should be investigated in the future.

## Authors' contributions

CFC designed the study, wrote the paper, imported animals, and performed the statistical analysis. DG and NZH supervised, organized and carried out the data collection. YPL and AB participated in the design of the experiment, commented on drafts of the paper. MTB participated in the coordination and design of the study, exported animals, discussed the interpretation of the results and commented on drafts of the paper.

## References

[B1] Bordas A Mérat P Effects of the naked-neck gene on traits associated with egg laying in dwarf stock at two temperatures Br Poult Sci 1984 25 195 207 10.1080/00071668408454858 6733551

[B2] Deeb N Cahaner A Genotype-by-environment interaction with broiler genotypes differing in growth rate, 1. The effects of high ambient temperature and necked-neck genotype on lines differing in genetic background Poult Sci 2001 80 695 702 11441834 10.1093/ps/80.6.695

[B3] Chen CF Bordas A Gourichon D Tixier-Boichard M Effect of high ambient temperature and naked neck genotype on performance of dwarf brown-egg layers selected for improved clutch length Br Poult Sci 2004 45 346 354 10.1080/00071660410001730842 15327121

[B4] Deeb N Cahaner A The effects of the naked neck genotypes, ambient temperature, and feeding status and their interactions on body temperature and performance of broilers Poult Sci 1999 78 1341 1346 10536779 10.1093/ps/78.10.1341

[B5] Horst P Rauen HW Khoo TH Significance of the naked neck gene (Na gene) in poultry breeding in the tropics Proceedings of the 7th European Poultry Conference: 1986; Paris, France 1986 24 28

[B6] Bordas A Mérat P Egg production performances of hens of the NaNa (homozygous naked neck), Nana+ (heterozygous) and na+na+ (normal plumage) genotype from a brown-egg dwarf (dw) line submitted to high constant temperature or to high temperature with periodic fluctuations Arch Geflugelk 1992 56 22 27

[B7] Mérat P Potential usefulness of the Na (Naked Neck) gene in poultry production World's Poult Sci J 1986 42 124 142 10.1079/WPS19860010

[B8] Mérat P The sex-linked dwarf gene in the broiler chicken industry World's Poult Sci J 1984 40 10 18 10.1079/WPS19840002

[B9] Chen CF Tixier-Boichard M Estimation of genetic variability and selection response for clutch length in dwarf brown-egg layers carrying or not naked neck gene Genet Sel Evol 2003 35 219 238 10.1051/gse:2003005 12633534 PMC2732696

[B10] Chen CF Tixier-Boichard M Correlated response to long-term selection for clutch length in dwarf brown-egg layers carrying or not the naked neck gene Poult Sci 2003 83 709 720 10.1093/ps/82.5.709 12762391

[B11] Tixier-Boichard M Boitard M Coquerelle G Mérat P Genetic improvement of clutch length in dwarf brown-egg layers: additional selection response with the naked neck gene Proceedings of the 20th World's Poultry Congress, 2–5 September 1996; New Delhi, India 1996 1 453 458

[B12] Bordas A Mérat P Sergent D Ricard FH Influence of the Na (naked neck) gene on growth, feed consumption and body composition of chicken according to environmental temperature Ann Génét Sél Anim 1978 10 209 231 10.1051/gse:19780205

[B13] Besbes B Ducrocq V Foulley JL Protais M Tavernier A Tixier-Boichard M Beaumont C Box-Cox transformation of egg-production traits of laying hens to improve genetic parameter estimation and breeding evaluation Livest Prod Sci 1993 33 313 326 10.1016/0301-6226(93)90010-F

[B14] SAS® Institute Inc SAS/STAT® User's Guide, Version 6.12. Cary, NC, USA 1999

[B15] Groeneveld E VCE4 User's Manual. Version 1.0 1996 Neustadt, Germany: Institute of Animal Husbandry and Animal Behavior

[B16] Groeneveld E PEST User's Manual 1990 Neustadt, Germany: Institute of Animal Husbandry and Animal Behavior

[B17] Cahaner A Deeb N Gutman M Effect of the plumage-reducing naked-neck (Na) gene on the performance of fast-growing broilers at normal and high ambient temperatures Poult Sci 1993 72 767 775

[B18] Deaton JW Reece FN Lott BD Effect of differing temperature cycles on broiler performance Poult Sci 1984 63 612 615 6728762 10.3382/ps.0630612

[B19] Keshavarz K Nakajima S The effect of dietary manipulations of energy, protein, and fat during the growing and laying periods on early egg weight and egg components Poult Sci 1995 74 50 61 7899213 10.3382/ps.0740050

[B20] Mathur PK Horst P Methods for evaluating genotype × environment interactions illustrated with laying hens J Anim Breed Genet 1994 111 265 288 10.1111/j.1439-0388.1994.tb00467.x 21395779

